# Validation of Wistar-Kyoto rats kept in solitary housing as an animal model for depression using voxel-based morphometry

**DOI:** 10.1038/s41598-024-53103-2

**Published:** 2024-02-13

**Authors:** Takanobu Yoshii, Naoya Oishi, Yasutaka Sotozono, Anri Watanabe, Yuki Sakai, Shunji Yamada, Ken-Ichi Matsuda, Masamitsu Kido, Kazuya Ikoma, Masaki Tanaka, Jin Narumoto

**Affiliations:** 1 Department of Psychiatry, Graduate School of Medical Science, Kyoto Prefectural University of Medicine, 465 Kajii-cho, Kamigyo-ku, Kyoto, 602-8566 Japan; 2 Medical Innovation Center, Kyoto University Graduate School of Medicine, 53 Shogoin Kawahara-cho, Sakyo-ku, Kyoto, 606-8507 Japan; 3 Department of Orthopaedics, Graduate School of Medical Science, Kyoto Prefectural University of Medicine, Kyoto, Japan; 4 Department of Neural Computation for Decision-Making, ATR Brain Information Communication Research Laboratory Group, Kyoto, Japan; 5 Department of Anatomy and Neurobiology, Graduate School of Medical Science, Kyoto Prefectural University of Medicine, Kyoto, Japan; 6 Kyoto Prefectural Rehabilitation Hospital for Mentally and Physically Disabled, Naka Ashihara, Johyo, Kyoto 610-0113 Japan

**Keywords:** Diseases of the nervous system, Psychosis, Brain, Psychology

## Abstract

Major depressive disorder is a common psychiatric condition often resistant to medication. The Wistar-Kyoto (WKY) rat has been suggested as an animal model of depression; however, it is still challenging to translate results from animal models into humans. Solitary housing is a mild stress paradigm that can simulate the environment of depressive patients with limited social activity due to symptoms. We used voxel-based morphometry to associate the solitary-housed WKY (sWKY) rat model with data from previous human studies and validated our results with behavioural studies. As a result, atrophy in sWKY rats was detected in the ventral hippocampus, caudate putamen, lateral septum, cerebellar vermis, and cerebellar nuclei (*p* < 0.05, corrected for family-wise error rate). Locomotor behaviour was negatively correlated with habenula volume and positively correlated with atrophy of the cerebellar vermis. In addition, sWKY rats showed depletion of sucrose consumption not after reward habituation but without reward habituation. Although the application of sWKY rats in a study of anhedonia might be limited, we observed some similarities between the regions of brain atrophy in sWKY rats and humans with depression, supporting the translation of sWKY rat studies to humans.

## Introduction

Major depressive disorder (MDD) is one of the most common psychiatric disorders. Globally, more than 264 million people of all ages suffer from depression^1^, and a meta-analysis of studies conducted in 30 countries from 1994 to 2014 reported that the aggregate prevalence of depression was 12.9%^2^. However, more than 60% of patients cannot achieve sustained remission with any single antidepressant drug therapy, and one-third of patients receive a diagnosis of treatment-resistant MDD^3^.

Voxel-based morphometry (VBM) is a well-established comprehensive analysis for structural brain magnetic resonance imaging (MRI)^4,5^. In human clinical studies, meta-analyses have already investigated common variations in MDD^6^. Still, the reported regions with significant atrophy have gradually shifted from the frontal areas, including the anterior cingulate cortex (ACC)^7,8^, parietal and limbic regions^9–11^ and the cerebellum^12^. Further, many people with bipolar disorder (BD) are misdiagnosed with MDD^13,14^, and reduced responsiveness to antidepressants may be due to veiled bipolar mood variations. A recent meta-analysis comparing MDD, BD, and healthy controls found atrophy in the dorsolateral prefrontal cortex, hippocampus, and cerebellum in patients with MDD^15^. However, additional studies investigating these changes are still needed, and animal experiments may offer one of the practical options.

Animal models have been applied for depression research, including social behaviours^16^. The Wistar-Kyoto (WKY) rat was initially established from Wistar rats as a normotensive control strain for spontaneously hypertensive rats^17^. WKY rats have since been used to assess a wide range of behavioural changes linked to symptoms seen in human MDD, including exaggerated stress responses, increased anhedonia, learned helplessness, novelty-induced hypophagia, neophobia, decreased locomotor activity, and increased anxiety, allowing for face validity as a depression model^18–21^. Further, WKY rats are considered a model of treatment-resistant depression because they are hyporesponsive to antidepressants^17,22,23^. Although predictive validity is still in discussion, this is important because of the similar response often observed in MDD patients.

Wistar rats have also been applied as an animal depression model combined with chronic variable stresses^24^. Wistar rats are highly distributed and used in many disease models (including surgery). Many depressed patients exhibit restricted social activity^25^, and there are significant associations between reduced social skills and depression^26^. Therefore, we suggest that an isolation paradigm in animal studies could mimic the typical environment of patients with depression. We were unable to confirm in the literature the statement of depressive behaviour due to isolation housing within ten days using Wistar rats. However, WKY rats within an isolation paradigm often exhibit enhanced depressive symptoms^27–31^.

The mechanisms underlying treatment resistance in patients with MDD remain unclear. Although several clinical studies have investigated this, the involvement of drug-naive patients presents challenges because the concepts of treatment resistance and drug naivety are incompatible. Conducting VBM in an animal model may be the first step in connecting the findings of animal and human studies.

We aimed to validate the similarities in atrophic brain regions between WKY rats kept in solitary housing (sWKY) and humans with MDD with an animal VBM study using MRI and compare the results with clinical MDD studies. Then, we examined the correlations between behavioural and morphological changes to validate sWKY rats as a model for depression.

## Results

### Animal body weight and total intracranial volume

A significant difference was observed in body weight (Wistar-pair: 381.79 ± 7.57 g, Wistar-single: 350.24 ± 9.46 g, sWKY: 241.37 ± 2.64 g;* F* = 104.88; *p* < 0.001) and Total intracranial volume (TIV), which was calculated using the MRI data (Wistar-pair: 2245.80 ± 34.60 mm^3^, Wistar-single: 2166.46 ± 42.54 mm^3^, sWKY: 2056.32 ± 27.00 mm^3^; *F* = 7.33; *p* = 0.002). Because we did not apply food control in this study, TIV is used as a covariate for analysis. Total number of rats: Wistar-pair: n = 14, Wistar-single: n = 13, sWKY: n = 13.

### Two open-field tests

**Figure 1:**
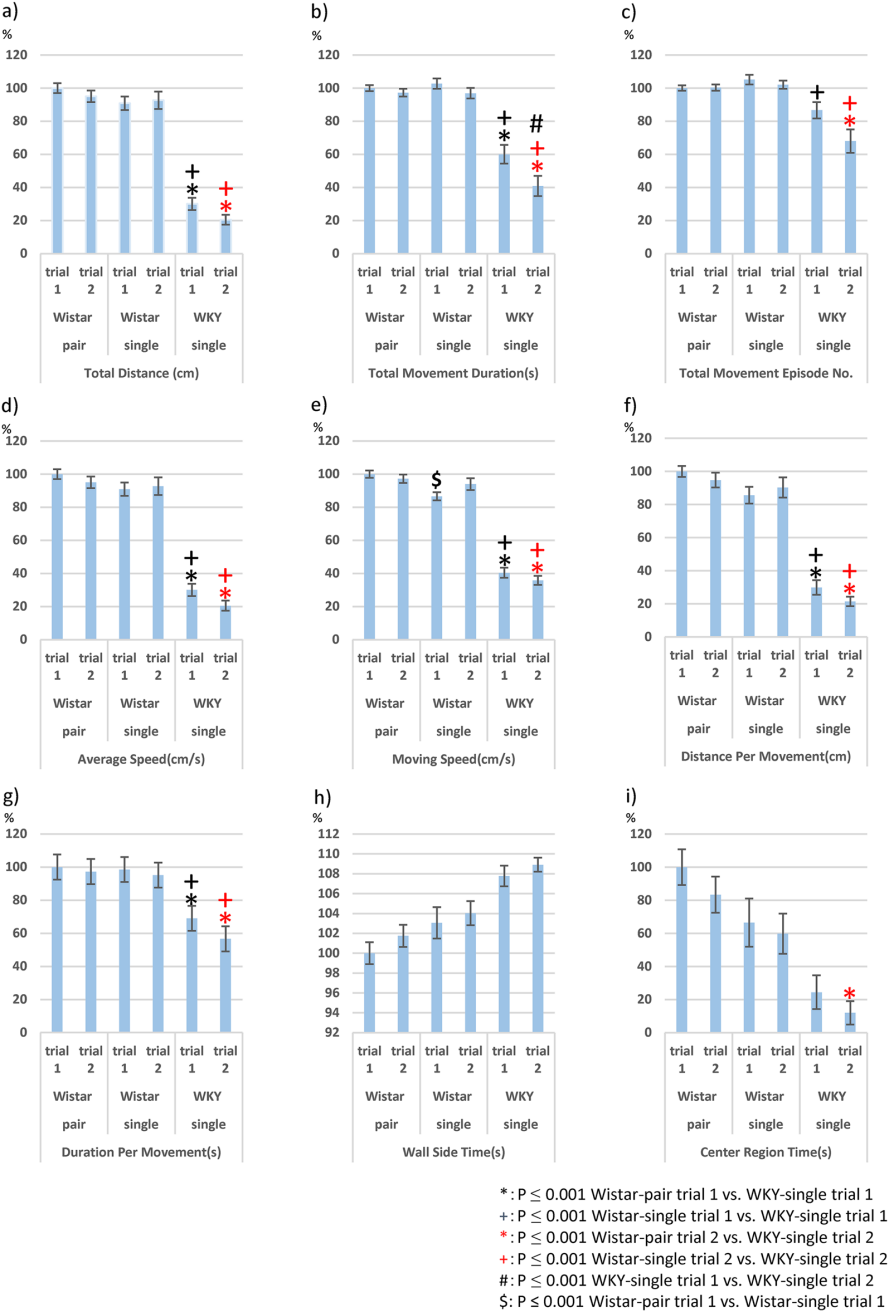
Behaviour Data in two OFT of Wistar-pair (n = 14), Wistar-single (*n* = 13) and Wistar-Kyoto-single (WKY-single, *n* = 13) rats: (**a**) total distance; (**b**) total movement duration; (**c**) total number of movement episodes; (**d**) moving speed; (**e**) duration per movement; (**f**) distance per movement; (**g**) average speed; (**h**) wall-side time; and (**i**) the total amount of time spent in the centre region. Percentage of the average of trial 1 of Wistar-pair rats was calculated, and bar plots were applied. Error bars indicate the standard error of means. Asterisks (*Wistar-pair vs WKY-single) and crosses (^+^Wistar-single vs WKY-single) indicate significance (*p < 0.001 and ^+^*p* < 0.001 repeated-measures general linear model (GLM) with Bonferroni correction with total intracranial volume (TIV) as a covariate). The asterisk and cross colour indicate the trial number of OFT, respectively (black cross and star: trial 1; red cross and star: trial 2). Between trials and housing environments (pair and single), we noted only very slight changes could be detected. In comparison between trials 1 and 2, WKY-single rats indicate a significant difference in total movement duration (^#^*p* < 0.001, **b**). We also noted the significant difference in moving speed (^$^*p* < 0.001, **e**) in comparison between Wistar pair and single rats in trial 1.

The locomotor activity of sWKY rats was disrupted after the initial phase (three days) and decreased further following an additional seven days in individual housing (Supp. Fig. S1). We tried measurement twice in animal groups (Wistar-pair: n = 14 Wistar-single: n = 13, sWKY-single: n = 13), and the comparison between trials 1 and 2shows animal sensitivity to moderate stress. We measured *behaviours* in OFT as follows: Total Distance (cm), Total Movement Durations (sec), Total Movement Episodes Numbers, Average Speed (cm/s), Moving Speed (cm/s), Distance Per Movement (cm), Duration Per Movement (sec), Wall side time (sec), Centre region time (sec), and time spent in blocks 1–25 respectively. Groups × *behaviours* (*F* (2, 33) = 30.356, *p* < 0.001) and *behaviours* (*F* = 4.242, *p* < 0.001) exhibited significant interactions; however, the interactions of Groups × *behaviours* × trials (*F* (2, 33, 1) = 1.110, *p* = 0.261), groups × trials (*F* (2, 1) = 2.41, *p* = 0.104), *behaviours* × trials (*F* (33, 1) = 0.076, *p* = 1.0), and trials (*F* = 0.037, *p* = 0.848) were not significant. In this study, to avoid type 1 error for multiple comparisons, we defined *p* ≤ 0.001 as significance. All locomotive behaviours (Total Distance (cm) (Fig. 1a), Total Movement Durations (sec) (Fig. 1b), Average Speed (cm/s) (Fig. 1c), Moving Speed (cm/s) (Fig. 1d), Distance Per Movement (cm) (Fig. 1e), Duration Per Movement (sec) (Fig. 1f) were significantly reduced in sWKY rats comparing with Wistar-pair and -single rats in both OFT trials (-g). Total movement episode No. also reached significance between Wistar-pair, -single rats and sWKY in the 2^nd^ trial, and between Wistar-pair and WKY in the 1^st^ trial. Anxiety-related behaviours (Wall side time (sec) (Fig. 1h), Centre region time (sec) (Fig. 1i)) did not reach significance except centre region time in the 2nd trial comparing between Wistar-pair and sWKY (Fig. 1i).

**Figure 2:**
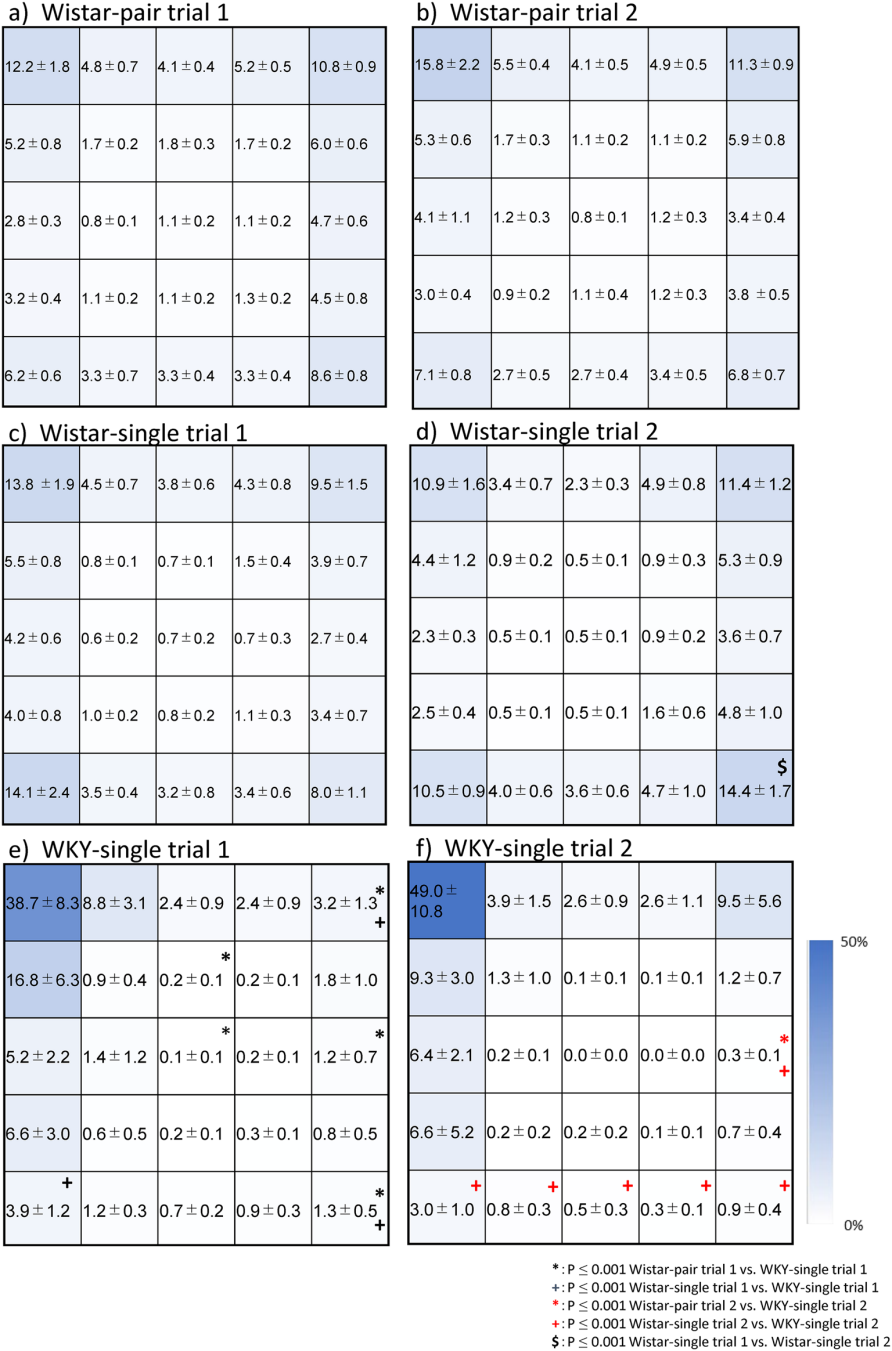
Block stay durations of Wistar-pair, Wistar-single, and WKY-single rats in the OFT trials 1 and 2. The figures respectively show (**a**) Wistar-pair rats in the OFT trial 1, (**b**) Wistar-pair rats in the OFT trial 2, (**c**) Wistar-single rats in the OFT trial 1, (**d**) Wistar-single rats in the OFT trial 2, (**e**) WKY-single rats in the OFT trial 1, and (**f**) WKY-single rats in the OFT trial 2. The numbers of block dimensions were presented in Supp. Fig. S2. Mean % of total ± SEM is calculated and described in the centre of the box and coloured with a blue colour scale (0–50%). In this figure, asterisks (*Wistar-pair vs WKY-single) and crosses (^+^Wistar-single vs WKY) indicate significance (i.e., *p < 0.001 repeated-measures general linear model (GLM) with Bonferroni correction with total intracranial volume (TIV) as a covariate). The Wistar-single group indicated significance only in time spent in block 25 in comparison between trials. In the 1st trial, the Wistar-pair stayed significantly longer in blocks 8 and 13 (centre zone) compared with WKY-single. Both pair and single Wister stayed corner significantly (Wistar-pair: block5, 25; Wistar-single: block5, 21, 25; **e**). In the 2nd trial, the significance in centre zone (block 8 and 13) in comparison between Wistar-pair and WKY was diminished, whereas Wistar-single stayed longer in blocks 20–25 than WKY (**f**). Time spent in block15 in 1st trial and 2nd trial of Wistar-pair and 2nd trial of Wistar-single were significantly longer than WKY-single. Block stay durations of WKY rats in the OFT trial 2. Time spent in block 1 significantly increased, whereas it decreased in block 6. The black cross was applied on the comparison between strains in this trial ^+^*p* < 0.001. WKY-single rats compared with Wistar did not flee to move blocks 15, 21–25: *p* < 0.001. Between trials, we rarely noted significance in time spent in blocks, although it was noted a significant difference in the time spent in block25 in Wistar-singles when comparing the first and second trial (^$^*p* < 0.001, **d**). Wistar-pair rats: n = 14, Wistar-single rats: n = 13, WKY-single rats: n = 13.

Between trials, no significant differences in behaviours and block-spent times were observed in the Wistar-pair group. Wistar-single indicated a significant increase in only time spent in block 25 in comparison between trials (Fig. 2d). sWKY indicated significantly lower moving speed in comparison between trials (Fig. 1b).

The author has been interested in the blocks where three strains spent time in the 2 OFT paradigm. The Wistar-single group indicated significance only in time spent in block 25 in comparison between trials. In the 1^st^ trial, Wistar-pair stayed long significantly in blocks 8 and 13 (centre zone) compared with sWKY (Fig. 2e). Both pair and single Wister stayed corner of the open field significantly (Wistar-pair: block5, 25; Wistar-single: block5, 21, 25) (Fig. 2e). In the 2nd trial, the significance in the centre zone (block 8 and 13) between Wistar-pair and sWKY was diminished, whereas Wistar-single stayed longer in blocks 20–25 than sWKY (Fig. 2f).

Block 15 seems to be irregular, and the time spent in block 15 in the 1st trial and 2nd trial of Wistar-pair and 2nd trial of Wistar-single were significantly longer than sWKY (Fig. 2e,f). Blocks 5, 10, 15, 20, and blocks 21, 22, 23, 24 have the same distance from the start position; it may just be that blocks 10 and 20 were not significant due to left/right preferences.

### Immobility time and low locomotion during forced swim test

**Figure 3:**
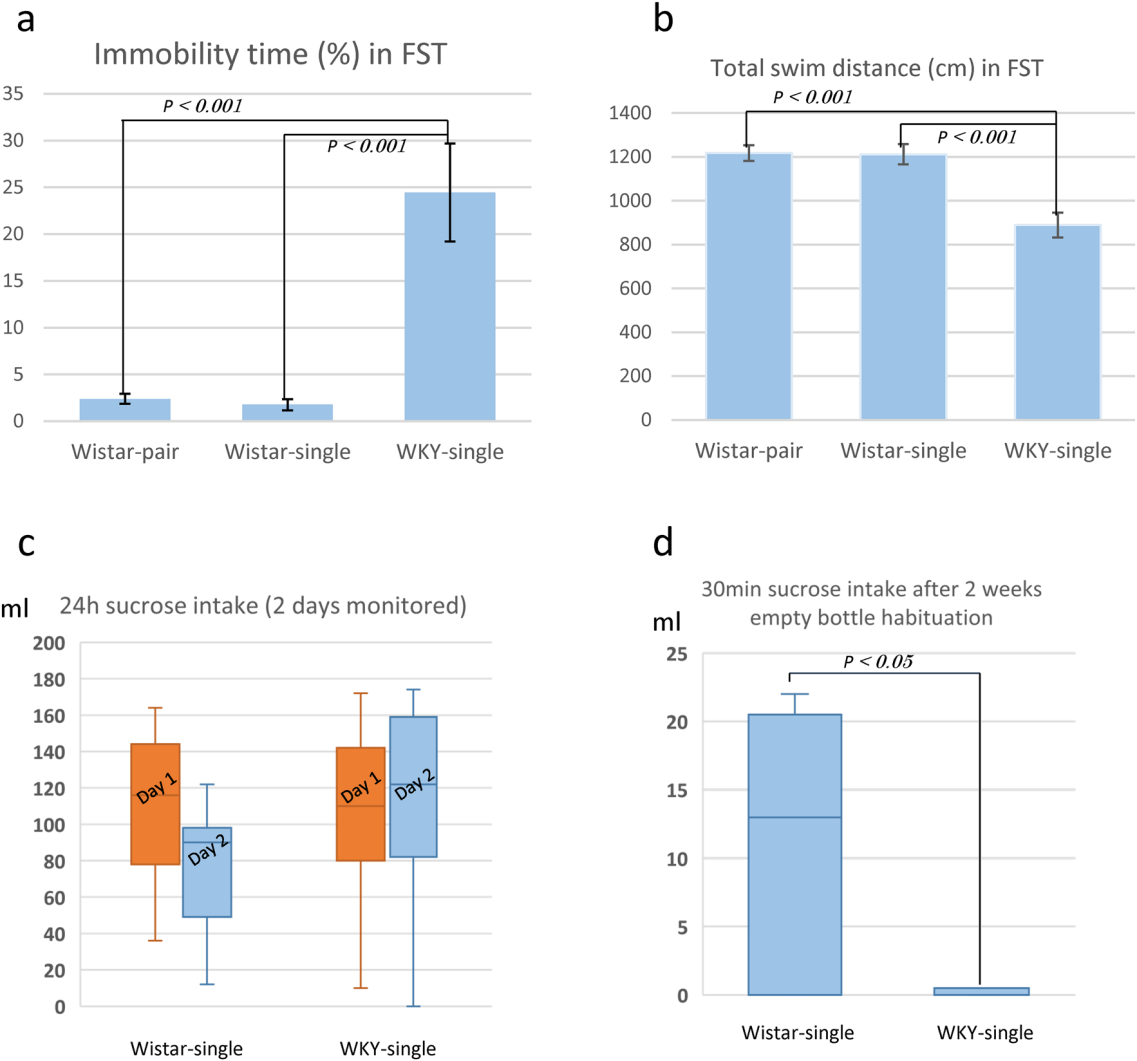
(**a**) Immobility time, defined as the per cent time spent immobile during the FST, was significantly higher in WKY-single rats (n = 13) than in Wistar-pair (n = 14) and single (n = 11) rats (t = 17.03; *p* < 0.001). (**b**) Total movement distance (cm) as a measure of locomotor activity during the FST was significantly shorter in WKY-single rats (n = 13) than in Wistar pair (n = 14) and single (n = 11) rats (t = 16.323; p < 0.001). (**c**) Consumption of sucrose for two days monitored. We investigated 2 days consumption after sucrose habituation (Wistar-single: n = 9 Day 1: 111.56 ± 42.35 ml, Day 2: 76.00 ± 11.39 ml, WKY-single: n = 9 Day 1: 105.78 ± 48.73 ml, Day 2: 114.00 ± 18.25 ml). Orange columns indicate Day 1, and blue indicate Day 2. We assigned no significant interaction and difference between strain and measurement day. Sucrose consumption after empty bottle habituation. Sucrose consumption was significantly reduced in WKY-single rats (Wistar: n = 6; 11.33 ± 3.95 ml, WKY-single: n = 6; 0.33 ± 0.817 ml, t = 2771, *p* < 0.05 Welch’s t-test).

Immobility time was compared in both strains during the 6-min forced swim test (FST) and was assessed as a measure of disrupted locomotion, a depressive trait. Immobility time, or the per cent of time immobile, was significantly longer in sWKY-single rats than in Wistar rats (Wistar-pair: 2.40 ± 0.54%; Wistar-single: 1.77% ± 0.59%; sWKY-single: 24.45% ± 5.25%; *f* = 17.03; *p* < 0.001; Fig. 3a). Total movement distance, a measure of locomotor activity, was also significantly less in sWKY rats than Wistar rats (Wistar-pair: 1217.60 ± 35.97 cm; Wistar-single: 1211.84 ± 46.42 cm; sWKY: 889.55 ± 56.56 cm;* f* = 16.327; *p* < 0.001; Fig. 3b). sWKY rats thus exhibited reduced locomotor activity and more immobility compared to Wistar rats during the initial phase of this study. The data from two Wistar rats were excluded due to camera failure (Wistar-pair: n = 14, Wistar-single: n = 11, sWKY: n = 13). To avoid type 1 error for multiple measurements (including OFT), we defined p ≤ 0.001 as significance.

### Sucrose consumption test

Sucrose consumption is a well-established indicator of anhedonia and has been used in many studies. However, previous reports on sucrose consumption/preference in sWKY rats in comparison with Wistar were still inconsistent: significant^32–34^ or not^35–37^. We initially investigated this after habituation with sucrose; however, we observed no significant differences in 2 day measurement between sWKY and Wistar rats (Day1: Wistar: n = 9, 111.56 ± 42.35 ml, sWKY: n = 9, 105.78 ± 48.73 ml; Day2: Wistar: 76.00 ± 11.39 ml, sWKY: 114.00 ± 18.25 ml, no significant interaction between strain and measurement day; strain: *f* = 0.951, *p* = 0.344, Day: *f* = 0.983 *p* = 0.336, Fig. 3c). There has been a report which mentions the retainment of WKY rats for anticipation of sucrose^33^ and we hypothesised that sucrose intake as a reward-seeking response would be maintained under the higher expectancy for rewards in sWKY rats. We, therefore, conducted a follow-up study using a protocol to maintain low expectancy for reward: habituation only to empty bottles. As a result, sucrose consumption was significantly reduced (Wistar: n = 6; 11.33 ± 3.95 ml, sWKY: n = 6; 0.33 ± 0.817 ml, t = 2,771, *p* < 0.05 Welch’s t-test, Fig. 3d). We consider that sWKY rats only show a decrease in seeking rewards in the low expectancy-reward situation. We also assume that sWKY rats may take more time to gain recognition as a reward concept. This sucrose consumption study did not use the same animals used in OFT and FST. Thus, the data were considered independent from other behavioural studies, and the significance level was set at P < 0.05.

### Results of voxel-based morphometry and voxel-based correlation analysis

**Figure 4:**
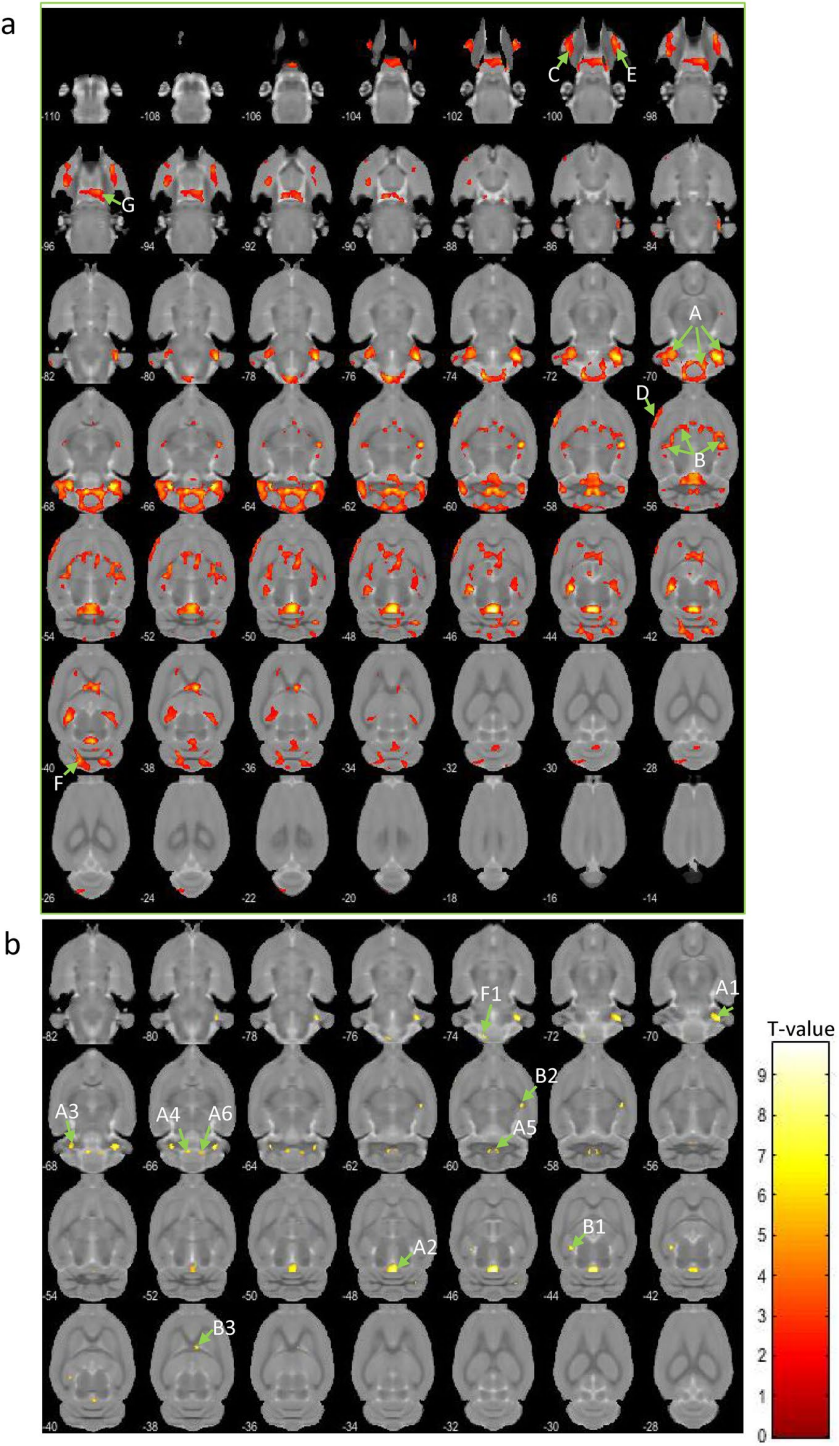
(**a**) Axial brain slices showing strain differences resulting in significant atrophy (WKY-single < Wistar-single) of part of the cerebellum (clusters A and F), the bilateral hippocampus to the septum (cluster B), bilateral amygdala (clusters C and E), insular cortex (cluster D), and pituitary gland (cluster G). Height level, *p* < 0.001; cluster family-wise error (FWE) corrected, *p* < 0.05. (**b**) Axial brain slices showing atrophy (WKY < Wistar) under the stricter threshold (*p* < 0.05; FWE-corrected; minimum voxel size: 19). With this threshold, atrophic clusters were present in the cerebellar nuclei (A1 and A3–6), cerebellar vermis (A2 and F1), and parahippocampus (B1–3). The letters used are aligned with the cluster identification labels from the intermediate threshold results. Colour bar units represent *t*-scores.

Voxel-based morphometry (VBM) was performed after the MRI acquisition. We carried out a VBM comparison between Wistar-pair (n = 14) and Wistar-single (n = 13) rats; however, no significant suprathreshold clusters were detected (data not shown). In this study, we aimed at both simplifying the experimental paradigm and simulating the human patient's environment and employed isolation as sham stress, limiting the study to comparisons between strains. Thus, VBM analysis in this study focused on the differences between Wistar-single (n = 13) and sWKY (n = 13). The VBM results from axial slices are presented in Fig. 4a,b. No significant hypertrophic (sWKY > Wistar) clusters were detected (height level, *p* < 0.001; cluster family-wise error (FWE) corrected, *p* < 0.05), although there were seven significant atrophic (sWKY < Wistar) clusters (clusters A–G; Fig. 4a; Table 1): the cerebellum (cluster A); the bilateral ventral hippocampus, extending to the septum (cluster B); the bilateral basolateral amygdala (clusters C and E); the left secondary somatosensory cortex, extending to the insular cortex (cluster D); and the pituitary gland (cluster G). With a stricter threshold (*p* < 0.05; FWE-corrected; minimum voxel size: 19), the following clusters remained (Fig. 4b; Table 2): the cerebellar nuclei (clusters A1, A3, A4, and A5), the rostral cerebellar vermis (cluster A2), the caudal cerebellar vermis (cluster F1), the left ventral hippocampus (cluster B1), the right caudate putamen (cluster B2), and the right lateral septum (cluster B3).

**Table 1:** Significant clusters of VBM using the moderate threshold.

	Cluster	Cluster-size	Peak			Estimated area
	P (few corrected)		T value	Z value	P (uncorrected)	
A	0.000	20,858	9.77	6.08	0.000	Cerebellum
B	0.000	10,039	8.07	5.51	0.000	Bilateral hippocampus and septum
C	0.008	1419	6.3	4.75	0.000	lt basomedial amygdala
D	0.012	1278	6.23	4.72	0.000	lt insular cortex
E	0.020	1135	6.04	4.63	0.000	rt basomedial amygdala
F	0.034	982	5.98	4.6	0.000	rt cerebellar caudal vermis
G	0.000	2381	5.9	4.56	0.000	Pituitary

*Lt* left, *rt* right.

**Table 2:** Significant clusters of VBM using the conservative threshold.

	Cluster	Cluster-size	Peak			Estimated area
	P (FWE corrected)		T value	Z value	P (FWE corrected)	
A1	N/A	488	9.77	6.08	0.000	rt cerebellar lateral nucleus
A2	N/A	629	9.76	6.08	0	cerebellar rostal vermis
A3	N/A	85	8.3	5.59	0.001	lt cerebellar lateral nucleus
A4	N/A	134	7.6	5.32	0.004	lt cerebellar internal nucleus
A5	N/A	46	6.63	4.91	0.021	rt cerebellar internal nucleus
A6	N/A	35	6.46	4.83	0.029	rt cerebellar medial nucleus
B1	N/A	94	8.07	5.51	0.001	lt ventral hippocampus
B2	N/A	82	7.47	5.27	0.004	rt caudate putamen
B3	N/A	39	7.2	5.16	0.007	rt finbria
F1	N/A	70	7.65	5.34	0.003	rt caudal cerebellar vermis

**Figure 5:**
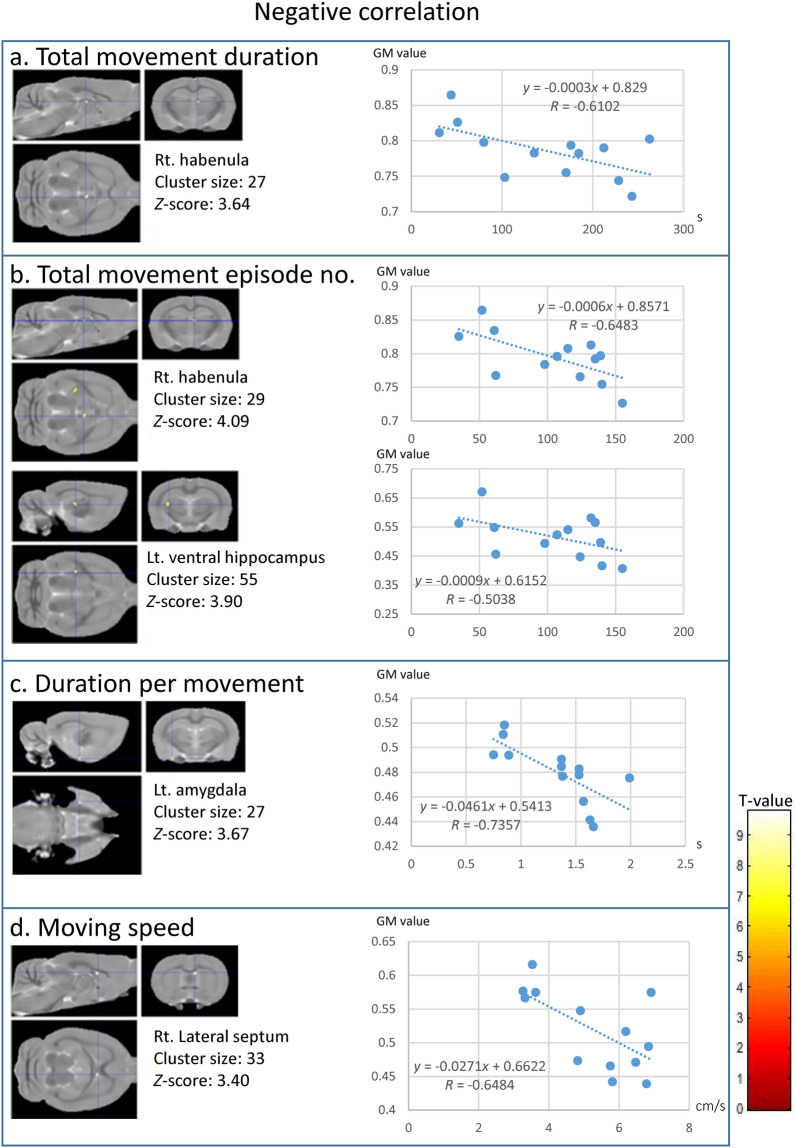
Clusters with negative correlations in Wistar-Kyoto-single (WKY-single) rats (*n* = 13). A threshold level of *p* < 0.001, uncorrected for multiple comparisons, and a minimum of 19 voxels in a cluster were applied. (**a**) Total movement duration. (**b**) Total number of movement episodes. (**c**) Duration per movement. (**d**) Moving speed. The negatively correlated clusters were in the right habenula, left ventral hippocampus, left amygdala, and right lateral septum. The adjacent graphs show the correlation between a given behaviour and the grey matter value inside the clusters detected by the voxel-based correlation analysis. Colour bar units represent *t*-scores.

**Figure 6:**
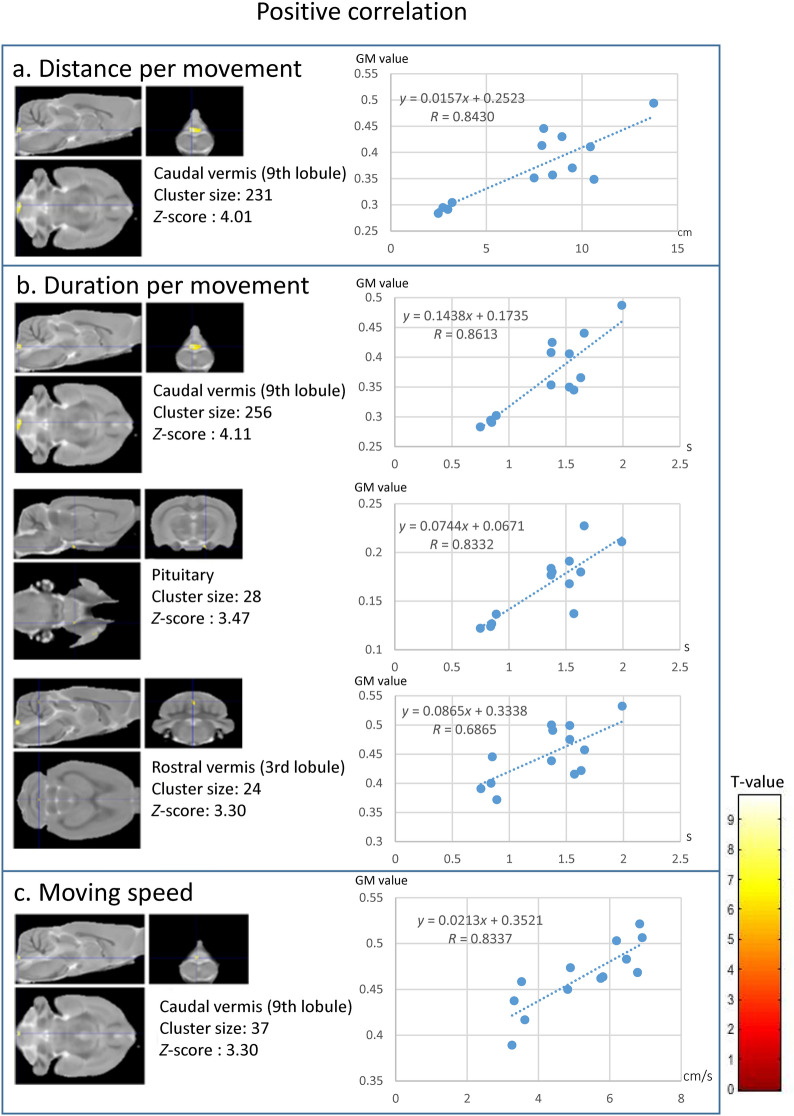
Clusters with positive correlations in Wistar-Kyoto-single (WKY-single) rats (*n* = 13). A threshold level of *p* < 0.001, uncorrected for multiple comparisons, and a minimum of 19 voxels in a cluster were applied. (**a**) Distance per movement. (**b**) Duration per movement. (**c**) Moving speed. The positively correlated cluster sets were located in the caudal vermis, rostral vermis, and pituitary gland. The adjacent graphs show the correlation between a given behaviour and the grey matter value inside the clusters detected by the voxel-based correlation analysis. Colour bar units represent *t*-scores.

We also performed a voxel-based correlation analysis between the parameters from the second OFT, except for time spent in each block and grey matter concentration in sWKY rats. We observed clusters in different brain areas with negative correlations between grey matter volume and certain behaviours typically studied in depression research. We observed negative correlations between total movement duration and total number of movement episodes in right habenula clusters (Fig. 5a,b); between distance per movement and clusters in the left amygdala (Fig. 5c); total number of movement episodes and clusters in the left ventral hippocampus (Fig. 5b); and movement speed and clusters in the right lateral septum (Fig. 5d). On the other hand, clusters with positive correlations were detected in the cerebellar vermis and pituitary gland: a cluster in lobule 9 in the caudal vermis was positively correlated with distance per movement (Fig. 6a), duration per movement (Fig. 5b), and moving speed (Fig. 6c); a cluster in lobule 3 in the rostral vermis correlated with duration per movement (Fig. 6a); and a cluster in the pituitary gland correlated with duration per movement (Fig. 6b). However, no significant cluster was found to correlate grey matter with wall-side time, per cent of time in the centre region, or total time in the centre region.

## Discussion

VBM revealed significant atrophy in the following brain areas of sWKY rats in comparison with Wistar-single rats: the left ventral hippocampus, the right caudate putamen, the right lateral septum, the cerebellar vermis, and the cerebellar nuclei. According to a recent clinical meta-analysis^15^, the VBM results comparing sWKY and Wistar-single rats have some similarities to those in human clinical MDD studies. However, the largest atrophic cluster was observed in the cerebellum. In the present study, direct comparisons between Wistar-pair and Wistar-single showed only minor behavioural and morphological changes due to VBM, which were not significant. Because of these results, we assume that it is not a major problem to simplify the discussion by only comparing single-housed Wistar and sWKY rats in the present study. The behavioural results of the two OFT trials and the FST indicate behavioural characteristics of sWKY rats reducing locomotion and escape activity. Voxel-based correlation analysis revealed areas correlated with escape behaviour in the second OFT after seven days of isolation. We observed negative correlations in the hippocampus, which agree with the results of a previous animal study^38^, as the right habenula, and a positive correlation in the cerebellar vermis.

Although changes in behaviour in OFT due to changes in the housing environment cannot be completely ruled out, we believe that the results are largely influenced by strain differences. There is little difference as far as direct behavioural comparisons between Wistar-pair and Wistar-single are concerned (only Movement speed in the first OFT is significant). However, the areas where there are significant differences at the block stay points are different when compared to WKY. In comparison with sWKY, the Wistar pair spent significantly more time in the centre (time spent in blocks 8 and 13 of trial 1 and the entire centre of trial 2), whereas the Wistar single seemed to spend more time in the corners and distant wall sides. Direct comparison between Wistar-pair and Wistar-single shows no significant difference except for moving speed in the 1st trial.

On the other hand, sWKY showed decreased sucrose consumption only without reward habituation. Although the application of WKY rats for an animal model of anhedonia in depression might be cautious, our VBM results indicate similar anatomical changes in sWKY rats as in human depression. Thus, we consider that sWKY rats are suitable for their use as a depression model.

Surprisingly, the most significant atrophic cluster was detected in the cerebellum with a moderate threshold. The clusters observed in the cerebellar nuclei and vermis in the 3^rd^ and 9^th^ lobules remained after the stricter threshold was applied. A recent meta-analysis reported an atrophic cluster in the cerebellum of MDD patients^12^, and other studies have suggested cerebellar involvement in modulating various aspects of mood and cognition^39–41^. Hypertrophy has been observed in the cerebellum of depressed patients after successful treatment with electroconvulsive therapy^42^ as in the cerebellar vermis after the use of chronic antidepressant medication^43^. Another recent meta-analysis detected a hypertrophic cluster in the cerebellar vermis in BD patients relative to MDD patients^15^. In addition, positive correlations between atrophic clusters and certain behaviours were detected in the vermis (9^th^ lobule), although we could not rule out that the locomotor dependent variables are likely highly correlated with each other and, as a result, would be accounting for the same variance. However, we could not confirm positive correlations with other atrophic brain regions, such as the hippocampus. The cerebellar vermis is included in the cerebrocerebellum and receives target projections from motor areas^44^. According to our OFT results, atrophy in the vermis may lead to disrupted escape activity. Cerebellar atrophy could lead to behavioural disturbances and inability to perform in the pursuit of novelty, even in the presence of motivation, although we could not rule out that sWKY rats could show a fear response. The mechanism of cerebellar atrophy is still unclear, and we cannot rule out the possibility that reduced cerebellum volume was secondary to the effects in other mood-regulating brain regions. However, we detected the largest atrophic cluster in the cerebellum, and the cerebellum’s role in emotional processing^45^ is still being discussed. Therefore, the cerebellum’s contribution to depressive symptoms should be investigated further.

We observed a significant cluster extending from the hippocampus to the septal region, somewhat including the thalamus (B in Fig. 2a), with a moderate threshold. Only 3 clusters in the para-hippocampus, including habenula (B1-3 in Fig. 2b), remained after the stricter threshold was applied. A previous animal study reported negative correlations between locomotion and grey matter concentration in the ventral hippocampus^38^. Because the hippocampus is a common region of interest in MDD research, as supported by a recent meta-analysis^15^, the atrophy observed in our model’s hippocampus and parahippocampal areas indicates similarity to human studies. However, WKY rats have been reported to be vulnerable to stress^17^, and differential roles of the ventral (stress, emotion) and dorsal (cognition) hippocampi have been discussed^46,47^, so the atrophy we observed might be stress-related.

Our data demonstrate a negative correlation between escape behaviour and the volume of the right habenula, although it was possible that the other locomotor variables are closely related and could explain the same variation. The habenula and its effect on escape activity should be investigated further. It has been proposed that the lateral habenula could systematically learn to expect an adverse outcome, and frequent neural firing may lead to a state of continuous disappointment and hopelessness^48^. A significant volume reduction in the habenula of depressive patients has also been reported^49^, and the habenula is considered a critical region in MDD research^50^. Ketamine-induced metabolic reduction in the right habenula of treatment-resistant depression patients has been reported^51^, and the habenula may be a potential target for future research on treatment-resistant depression.

Brain atrophy observed in the right lateral septum neighbouring the fimbria remained when the stricter threshold was applied, and a negative correlation with escape behaviour was detected. Although the septal area is rarely described in clinical depression studies using MRI, previous animal studies reported that the septal nuclei are involved in stress responses^52^. In addition, lesion studies of the septum reported aggressive behaviours^53,54^. Patients with clinical MDD exhibit ambivalence, lost motivation, reduced locomotor activity, and increased impulsivity and irritation. Further study is warranted to clarify whether the atrophy that extends from the ventral hippocampus to the septum contributes to the complex symptoms observed in MDD.

Only two clusters, including the amygdala, were detected using the moderate threshold, although a negative correlation to movement duration was seen in the left amygdala. Some volumetric studies in MDD patients have reported increased amygdala volumes^55,56^, whereas others have reported reduced volumes^57,58^. A meta-regression analysis in first-episode MDD patients showed negative correlations between the right amygdala volume and Hamilton Depression Rating Scale score^10^. In another recent study, patients with treatment-resistant MDD exhibited blunted amygdala activity during a facial recognition task^59^. Further research is thus warranted to determine the relationship between amygdala volume and treatment-resistant MDD.

Our results indicated atrophy from the secondary somatosensory cortex extending to the insular cortex, although this did not remain after the stricter threshold was applied. Therefore, we cannot determine any major contribution of this site to MDD. However, a human study using VBM analysis^60^ and some meta-analyses^7,10,11^ reported insular cortex atrophy in MDD patients, and our VBM data seem consistent with these studies. Our voxel-based correlation analysis could not detect a significant cluster in the somatosensory or insular cortices. No correlation between these areas and behaviour could be confirmed in sWKY rats. Insular cortex atrophy is commonly observed in many psychiatric disorders^61^, so analysis of this region in post-mortem brain studies may help to elucidate genetic spectra across various psychiatric disorders^62^, including MDD.

To the best of our knowledge, a reduction in pituitary gland volume has not previously been reported in WKY rats. However, it has been demonstrated that isolation paradigms elevate plasma ACTH levels without changes in corticosterone levels^27^, and we cannot rule out the possibility that isolation exhausted pituitary ACTH in WKY rats, resulting in atrophy. However, our study showed that pituitary atrophy was positively correlated with locomotion behaviour, so future studies must determine whether this atrophy is a biomarker of treatment resistance.

OFT has been criticised as an anxiety-detecting measure^63^. It has been discussed that immobility during the FST is not indicative of despair but a coping benefit from learning and memory to promote behavioural adaptation and survival^64^. These may also contain other behavioural aspects, i.e., energy savings. Thus, we cannot rule this possibility out when interpreting the enhanced anxiety and preference for passive survival coping strategies in sWKY rats; however, OFT and FST have been used to assess psycho-motor impairment as a depressive phenotype^24^, and our data simultaneously demonstrated low locomotor activity during both the FST and OFT. Since we consider OFT to be one of the least stressful behavioural measurements to avoid acute brain morphological changes, we selected OFT as a behavioural measurement immediately before MRI acquisition. OFT would not relate to survival, and our behavioural results thus show reduced locomotor behaviour in neutral and threatening situations. We, therefore, assumed that our results in OFT were not entirely due to stress coping. It has also been discussed that reduced locomotor activity in inescapable environments is more associated with reduced novelty and stress responses^65^. On the other hand, there are limitations to this study; the behavioural data may not be normally distributed and the use of parametric tests in this study may have reduced statistical power. In addition, the influence of the first FST on the second OFT cannot also be excluded.

In this study, we observed a significant decrease in sucrose consumption only under the protocol without habituation to sucrose, whereas we observed no significant change after sucrose habituation. It should be ruled out that reward expectancy would be kept low in WKY rats without habituation to sucrose, and that the acquisition of the concept of reward in situations of low reward expectancy was delayed in WKY rats in a future study. We also could not rule out neophobia in sWKY rats. Thus, we should be cautious about applying the sWKY rat to anhedonia models. However, there are reports that sWKY shows sugar binging^37^ and retainment for sensitivity to palatable reward^33^. The co-occurrence between alcohol addiction and depression in humans has been discussed^66^ and it was suggested that the sWKY rat strain may represent a suitable model for studying the neurochemical mechanisms underlying depressive behaviour and increased alcohol consumption^67^. We should discuss that anhedonia may not be an essential condition for the depression model if there are abnormalities in reactivity to rewards like co-occurrence of addiction.

We housed rats individually and cannot completely rule out the effect of isolation on brain atrophy. However, there are few reports on microscopic morphological changes induced by social isolation (besides maternal separation) within the first two weeks; only one report describes olfactory bulb atrophy induced by isolation^68^. Although it has been proposed that a social isolation paradigm might have adverse effects on recovery interventions, such as inducing neurogenesis in the hippocampus following exercise^69,70^, no significant volumetric changes in the hippocampus were reported during two weeks of social isolation using the region of interest-volumetric MRI method^68^. In another depression model, the Flinders sensitive line, five weeks of isolation-induced the erasure of depression-like behaviour^71^. Whether isolation can be dismissed as stress in animal depression models is still an interesting question. We suspect that the effect of isolation stress in the first two weeks might be too small to affect brain morphology in animal depression models. However, we cannot determine the possibility that genetic differences might enhance environmental effects. It would be desirable to verify the behavioural differences in Wistar-Kyoto rats depending on the rearing environment in the future.

It should be noted that the influence of the first FST on both the second OFT and MRI data cannot be excluded. To minimise the effect on the MRI structural analysis, the FST should be performed at an early stage. In addition, FST is performed after the first OFT to eliminate the influence of the first OFT.

It has also been recommended to perform the behavioural tests during the dark phase of a 12- or 12-h light/dark cycle, when it is their most active time since their vision system is highly updated to the low-light condition^72^. Our behavioural experimental paradigms were carried out in the light phase, and the data might be influenced by the experimental timing. In addition, transportation days before 1st behavioural test (3 days) is not completely denied as a source of additional stress.

We used an ex vivo MRI protocol to achieve the quality necessary for VBM. However, ex vivo MRI measurements have been discussed as less preferable for structural brain studies due to potential displacement, disrupted brain tissue integrity, and deformations resulting in artifacts^73^. This method may cause a global shrinkage in brain volume (~ 10%), and damage is most notable in the cerebellum, olfactory bulb, and cortex^74,75^. Therefore, we cannot completely rule out any data noise caused by the fixation method. Spatially normalised images in VBM analysis should have a relatively high resolution in humans, and grey matter segmentation is not excessively confounded by partial volume effects^4^. Still, these assumptions may not hold in small animals. In vivo, MRI in small animals makes it difficult to prevent motion artefacts, and there are resolution disadvantages. In particular, it has been reported that the thalamus and hypothalamus can be more appropriately segmented using ex vivo MRI data^74^. An MRI study also notes that the midbrain hippocampus, thalamus, and cortex volume were unaffected by perfuse-fixation^75^. The essential data observed in parahippocampal regions and cerebellar nuclei in this study might have benefitted from using ex vivo MRI techniques.

Global VBM includes many voxel-by-voxel statistical analyses (over 1,000,000), so the established statistical threshold does not accurately capture its statistical detectability. Small-volume structures are difficult to detect with significance, and VBM cannot be used as a basis for rejecting sites detected by other methods. Numerous studies describe changes in various brain regions in animal depression models, especially in WKY rats. We cannot completely rule out potential psycho-physiological alterations and areas encoding depression-like behaviour that were not detected in these VBM results. However, apparent morphological changes are not expected in undetectable sites using VBM.

This study shows that sWKY rats demonstrated morphological similarities to MDD in humans. Correlations between the habeula and cerebellar vermis with inhibited escape behaviour were determined via voxel-based correlation analysis. Although the contributions of the cerebellum^39^ and habenula^50^ to MDD have been discussed, there is insufficient research and therapies directly targeting these areas. We believe that sWKY rats would be a good model for exploring the mechanisms of MDD and addressing therapeutic challenges.

## Materials and methods

### Animals

Eight-week-old male Wistar-pair (n = 14), Wistar-single (*n* = 13) and WKY-single (sWKY) rats (*n* = 13) were obtained from Charles River Laboratories Japan. (Yokohama, Japan). Initially, all rats were housed individually, maintained in all air-conditioned rooms (23 ± 1 °C) on a 12 h light/dark cycle (6:00–18:00 light), and fed food and water ad libitum. All experiments were approved by the Committee on Animal Research at Kyoto Prefectural University of Medicine and were conducted according to the animal care guidelines of the National Institutes of Health and ARRIVE guidelines^76^. We minimised the number of animals used and their suffering.

### Behavioural measurements and analysis

Two exposures to an open field were done to compare the anxiety-related and locomotive behaviour of the two rat strains. The first exposure trial provided a novel environment, while the second trial allowed us to observe exploratory motivation after the rats were kept in individual cages. The first exposures and behavioural measurements were conducted three days after the delivery date of the rats. Rats were then subjected to a 6-min forced swim test (FST). After seven additional days of being housed individually, the second open-field test (OFT) trial was performed (Supp. Fig. S1). Both are conducted during the light period: OFT: 10:00–11:00; FST: 12:00–13:00. We also followed the measurement of sucrose consumption as the indicator of anhedonia.

#### Open field test

Open-field tests (OFT) have been applied for the analysis of motivation and anxiety-related behaviours^77,78^. Animals were placed in an OFT apparatus (90 × 90 × 40 cm^3^) to explore the field freely for 10 min, and an overhead camera and tracking software recorded all movements (O’Hara and Co., Tokyo, Japan). The field was maintained at approximately 10 lx, and the animals were acclimated to the same brightness for approximately 30 min prior to the experiment. The surface and walls of the apparatus were cleaned with ethanol before and after each session. The locomotion data were obtained using this test apparatus packaged by O'Hara and Co., and the movement trajectories of the rats were captured by a CCD camera from above and converted to data using their software. To track movements, the apparatus was divided into 25 blocks, and blocks 7–9, 12–14, and 17–19 were defined as the centre region (Supp. Fig. S2). The total distance (cm), entire movement duration (s), the total number of movement episodes, average speed (cm/s), moving speed (cm/s), distance per movement (cm), duration per movement (s), wall-side time (s), per cent time in centre region (%), total time in centre region (s), and the time spent in each of the 25 blocks (s) were measured as behavioural parameters (simply referred to as *behaviours* in the former section). Statistical analysis was performed with a repeated-measures general linear model (GLM) with a covariate of total intracranial volume (TIV) and a modified multiple comparisons test using Bonferroni’s method and SPSS Statistics ver. 23.0.0.2 (IBM, Armonk, NY, USA).

#### Forced swim test

Forced swimming tests (FST) are used to assess depression^79^ and are sometimes assessed in combination with other behavioural tests, including OFT^80^. After the first OFT measurements, animals were placed in a 60 cm × 20 cm acrylic resin cylinder (O’Hara and Co.) containing water (height: 30 cm; temperature: 23 °C) for 6 min. The time spent immobile, which included floating and movements necessary for breathing, was assessed using video tracking software with two cameras (O’Hara and Co.). The total duration of swim movements was also measured. The freezing time was defined as the time during which the two cameras simultaneously recorded no movement. Two-tailed two-sample *t*-tests (Welch’s method) were applied for the statistical analysis.

#### Sucrose consumption test

Sucrose consumption behaviour has been used as an indicator of depression^81^. In this research, the sucrose consumption test is carried out using three methods. All bottle exchanges and measurements were carried out between 15:00 and 17:00. Initially, rats were housed individually for three days and habituated to two 2% sucrose bottles for one day. Next, rats were deprived of water for 24 h, then one tap water and 2%sucrose bottles were set for 24 h, and intake was measured as 1st-day consumption.

Second, rats were housed individually for three days, and two sucrose bottles were placed for 24 h for sucrose habituation; then, one tap water bottle and a 2%sucrose bottle were set for 24 h. Then bottles were exchanged, and sucrose consumption for 24 h as 2nd-day consumption was measured. In this case, water deprivation before the measurement was not applied.

We followed the disrupting reward expectancy protocol below. Rats were housed individually and habituated to two empty bottles for two weeks, then one tap water bottle and an empty bottle were set for 24 h. After this habituation protocol, rats were deprived of water for 24 h. Then, one tap water bottle and 2%sucrose bottle were placed, and a measurement of sucrose consumption for 30 min was carried out.

None of the animals used in the sucrose preference study were used for OFT/FST data or MRI data acquisition.

### Tissue preparation

After the behavioural measurements were taken, Wistar and WKY rats were deeply anesthetised with pentobarbital, then perfused and fixed with 4% paraformaldehyde. Rat skulls, including the brain, were removed and immersed in the same fixative overnight, then stored at 4 °C until MRI acquisition.

### Ex vivo brain MRI

We modified our previously established ex vivo brain MRI technique^82^ to acquire images of four rats simultaneously using a cradle (Rehabitech, Kyoto, Japan; Fig. 1g). Briefly, a Helmholtz small-volume coil (probe dimension: 108/63) was used for both radiofrequency excitation and signal detection in a 7 T Varian system (Agilent Technologies, Palo Alto, CA, USA). Fast spin-echo 3D volume was collected (TR: 2000 ms; TE: 20 ms; flip angle: 30°; FOV: 90 × 40 × 40 mm^3^), and a bias field inhomogeneity correction was applied to the acquired scans (acquisition matrix: 256 × 128 × 128; zero filled to 512 × 256 × 256; final voxel resolution: 0.176 × 0.156 × 0.156 mm^3^).

### Voxel-based morphometry (VBM) and voxel-based correlation analysis

MRI images were segmented to identify grey matter and spatially normalised with diffeomorphic anatomical registration using the exponentiated lie algebra (DARTEL) protocol in statistical parametric mapping software ver.12 (SPM12) and the in-house rat brain template and probabilistic maps were created in our previous study^82^. We also followed the VBM methods detailed previously^82^. Briefly, experimental brain MR images were denoised with a 3D non-local means filter, co-registered, and segmented to identify grey matter before spatial normalisation^82^. Jacobian determinants from the DARTEL procedure were calculated and used to modify the processed grey matter images. The images were resampled into 0.15 × 0.15 × 0.15 mm^3^ voxels and smoothed with an isotropic Gaussian kernel of 0.8 mm in the original space, which was full-width at half-maximum. For the group-level statistical analysis, we performed voxel-by-voxel two-sample *t*-tests across the whole brain by applying the GLM. TIV was set as a covariate in all analysed studies. The cluster extent thresholding correction method is used for multiple comparisons correction^83–85^ and the significance level was first set at a cluster height threshold of *p* < 0.001, with a determinant extent threshold of *p* < 0.05 corrected for family-wise error (FWE). We then used a more conservative peak level threshold of *p* < 0.05, corrected for multiple comparisons using the FWE correction method with a minimum cluster size of 19 voxels based on the number of expected voxels per cluster (*K* = 18.563).

Voxel-based correlation analysis was performed to determine the association between parameters (except the time spent in each block and brain volume in WKY rats). We cannot entirely dismiss brain volume alteration via the isolation paradigm. Therefore, we performed a correlation analysis between brain volume and the second OFT parameters parameter. We used only significant voxels detected by comparing the WKY and Wistar rats using the significant voxel map (*p* < 0.05, cluster FWE-corrected) as a brain mask. An uncorrected *p* < 0.001 with a minimum cluster size of 19 voxels was considered significant. Pearson’s *r* values were also calculated between parameters and mean grey matter voxel values of significant clusters.

### Supplementary Information

Supplementary Figures.

Supplementary Legends.

## Data Availability

The data that support the findings of this study are available from the corresponding author upon reasonable request.
