# Supplementary figures and images for: Validation of Wistar-Kyoto rats kept in solitary housing as an animal model for depression using voxel-based morphometry
Source: Sci Rep. 2024 Feb 13;14:3601. doi: 10.1038/s41598-024-53103-2 (PMC10864298; doi:10.1038/s41598-024-53103-2)

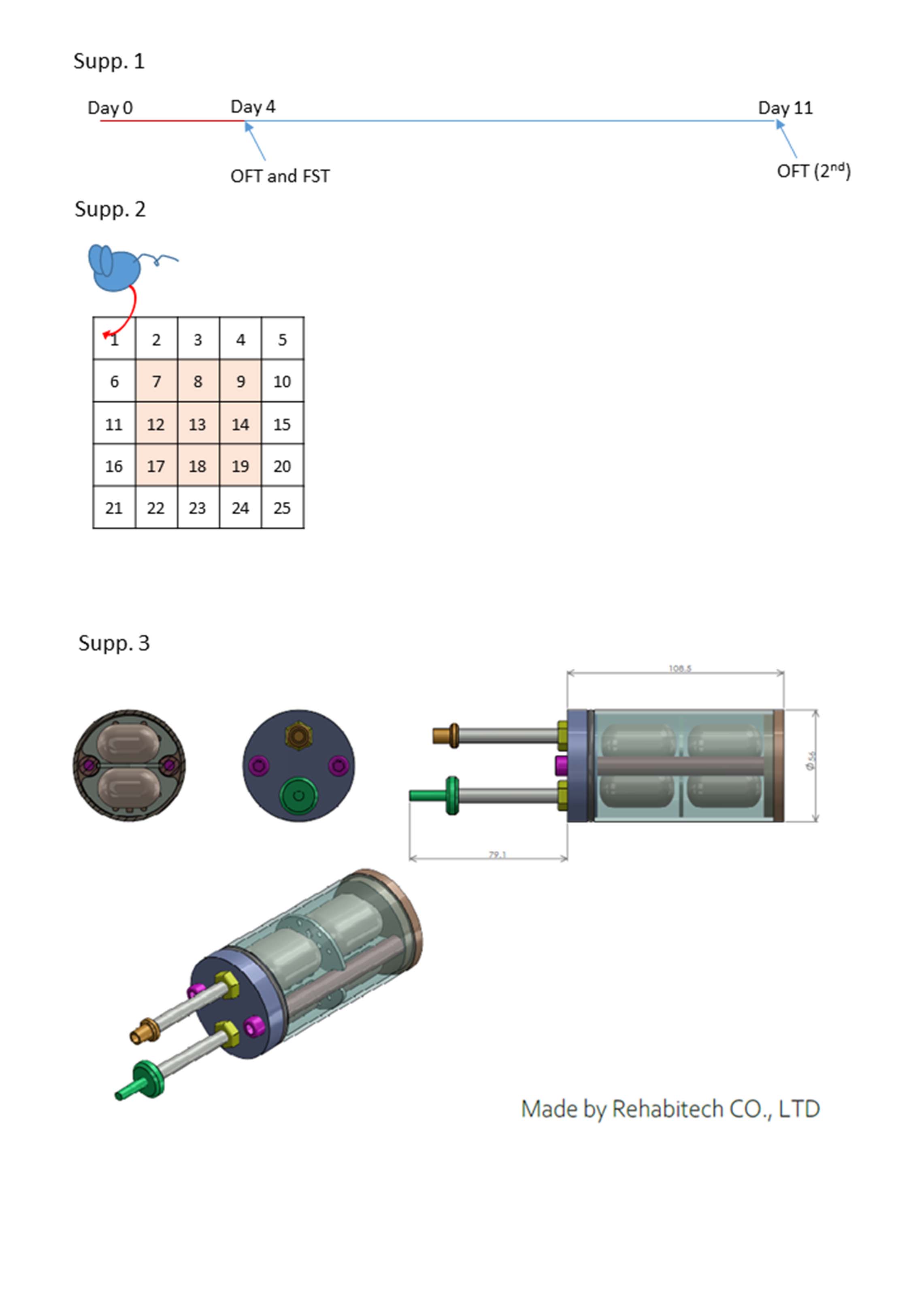

Supplement: Supplementary file 1 — Supplementary Figures. [file 41598_2024_53103_MOESM1_ESM.jpg]
