# Supplementary material for: Validation of Wistar-Kyoto rats kept in solitary housing as an animal model for depression using voxel-based morphometry
Source: Sci Rep. 2024 Feb 13;14:3601. doi: 10.1038/s41598-024-53103-2 (PMC10864298; doi:10.1038/s41598-024-53103-2)
Supplement: Supplementary file 2 — Supplementary Legends. [file 41598_2024_53103_MOESM2_ESM.docx]

Supplementary figure legends

1. Schedule of behavioural experiments, including two open-field test (OFT) trials and one forced swim test (FST). The rats were subjected to an OFT and FST on Day 4 and sacrificed on Day 11 after the second OFT. Then, Rat skulls, including the brain, were removed after fixation and stored at 4 °C until MRI acquisition.
2. The open-field apparatus. The open field was divided into 25 blocks, and behaviour was observed and measured using the software. Rats were placed in block 1 at the beginning of the test. Light orange shading indicates the centre region.
3. Dimensions of the cradle for magnetic resonance imaging, as described by

Rehabitech (Kyoto, Japan). An acrylic tube (106.5 mm × 86.5Φ) was divided into four segments. During image acquisition, skulls including the brain were set in the segments and fixed with parafilm. Fluorinert (3M Japan, Tokyo, Japan), a proton-free medium, was injected using an injection bulb to fill all segments. Air was removed using a separate bulb. This figure is described by Rehabitech Co,Ltd. And Copy right is permitted with a CC-BY 4.0　lisence.
